# Efficient and reproducible identification of mismatch repair deficient colon cancer: validation of the MMR index and comparison with other predictive models

**DOI:** 10.1186/1472-6890-13-33

**Published:** 2013-12-17

**Authors:** Patrick Joost, Pär-Ola Bendahl, Britta Halvarsson, Eva Rambech, Mef Nilbert

**Affiliations:** 1 Department of Pathology and Cytology, Helsingborg General Hospital, Helsingborg, Södra Vallgatan 5 SE-251 87, Helsingborg, Sweden; 2 Department of Pathology, Skane University Hospital, Clinical Science, Lund University, SE-221 85 Lund, Sweden; 3 Department of Oncology, Institute of Clinical Sciences, Lund University, SE-221 85 Lund, Sweden; 4 Aleris Medilab, Nytorpsvägen 28-32, SE-183 53 Täby, Sweden; 5 Clinical Research Centre, Hvidovre University Hospital, Copenhagen University, Kettegaards Allé 30, DK-2650 Hvidovre, Denmark

**Keywords:** Pathology, Colorectal cancer, Microsatellite instability, Mismatch repair, Prediction model

## Abstract

**Background:**

The identification of mismatch-repair (MMR) defective colon cancer is clinically relevant for diagnostic, prognostic and potentially also for treatment predictive purposes. Preselection of tumors for MMR analysis can be obtained with predictive models, which need to demonstrate ease of application and favorable reproducibility.

**Methods:**

We validated the MMR index for the identification of prognostically favorable MMR deficient colon cancers and compared performance to 5 other prediction models. In total, 474 colon cancers diagnosed ≥ age 50 were evaluated with correlation between clinicopathologic variables and immunohistochemical MMR protein expression.

**Results:**

Female sex, age ≥60 years, proximal tumor location, expanding growth pattern, lack of dirty necrosis, mucinous differentiation and presence of tumor-infiltrating lymphocytes significantly correlated with MMR deficiency. Presence of at least 4 of the MMR index factors identified MMR deficient tumors with 93% sensitivity and 76% specificity and showed favorable reproducibility with a kappa value of 0.88. The MMR index also performed favorably when compared to 5 other predictive models.

**Conclusions:**

The MMR index is easy to apply and efficiently identifies MMR defective colon cancers with high sensitivity and specificity. The model shows stable performance with low inter-observer variability and favorable performance when compared to other MMR predictive models.

## Background

Genomic destabilization is an intrinsic, tumor-promoting feature in most cancer cells. In colon cancer this is achieved through tumorigenic pathways related to chromosomal instability, CpG island methylation and mismatch repair (MMR) defects that cause microsatellite instability (MSI) [1]. The identification of MMR defective tumors provides prognostic information and identifies heritable cases linked to Lynch syndrome. MMR defective tumors are typically located proximal to the splenic flexure and are overrepresented among young patients (mean 45 years) in Lynch syndrome and older patients (mean 75 years) in sporadic cases [2-7].

Morphologic characteristics of MMR deficient tumors include an expanding tumor growth pattern, poor and mucinous differentiation, a solid/medullary growth pattern, lack of “dirty necrosis” and lymphocytic reactions such as peritumoral lymphocyte infiltration, Crohn-like reactions and presence of tumor-infiltrating lymphocytes (TIL) [6,8-10]. Increasing evidence suggests that the identification of MMR deficient tumors provides clinically relevant information, but universal MMR screening has not yet gained widespread application in clinical practice [11-13].

Our study focuses on the 20% of the non-hereditary colon cancers with somatic *MLH1* promoter methylation that are associated with a favorable prognosis and a suggested poor response to 5-fluorouracil-based regimens [11-18]. A prescreening procedure that identifies tumors with a high likelihood of MMR deficiency could be clinically valuable for institutions that have not implemented universal assessment of MMR status. Several predictive models aimed at identifying tumors with an increased likelihood of MMR deficiency have been established (Table 1) [19-24]. These models have predominantly focused on the identification of Lynch syndrome tumors, whereas the MMR index and the RERtest6 model were developed in series that had a substantial contribution from sporadic MMR deficient tumors [20,24,25]. For such models to be implemented in the routine histopathologic work-up, the assessment should be easy to apply, i.e. preferentially be based on factors that can be evaluated on standard sections, and reproducible. We validated the MMR index in an independent series of 474 colon cancers and provide data on the reproducibility and performance in comparison with 5 other MMR/MSI-predictive models.

**Table 1:** Summary of clinicopathologic features evaluated in the different models to predict MMR deficiency

**Model (reference)**	**MMR index [**[25]**]**	**MsPath [**[19]**,**[23]**]**	**PREDICT [**[22]**]**	**MSI probability score [**[21]**]**	**RERtest6 [**[20]**,**[24]**]**
**No. of variables**	7	6	6	7	6
**Sex**	Female	-	-	-	-
**Age (years)**	≥60	<50	<50	<50	-
**Tumor location**	Proximal	Proximal	Proximal	Proximal	Proximal
**Growth pattern**	Expanding	-	-	-	Expanding
**Dirty necrosis**	Lack of	-	-	Lack of	-
**Mucinous/signet-ring components**	≥10%	≥50% (including medullary carcinoma)	Any component	Any component	Amount in %
**Solid component**		-	-	-	Amount in %
**TIL**	≥7 TIL/10 HPF	≥5 TIL/HPF (10 HPFs searched)	≥ 5TIL/HPF (10 HPF searched)	≥2 TIL/mean of 5 HPF	≥4TIL/HPF
**Differentiation**	-	Poorly differentiated	-	Well or poorly differentiated	-
**Crohn-like reaction**	-	≥4 nodules/LPF	-	≥3 nodules/section	≥3 nodules/LPF
**Peritumoral lymphocytic reaction**	-	-	Banding of lymphocytes beyond advancing edge	-	-
**Increased stromal plasma cells**	-	-	>25% plasma cells/stromal immune cells	-	-
**Scoring system**	No score, 7-factor index, cut-off ≥4	Score: cut-off ≥1	Score: cut-off ≥2.5; “Simplified PREDICT”: no score, ≥2 features present	Score: cut-off ≥1 or ≥1.5	Score: cut-off <0.8
**Sensitivity**	92.3% (4 features of 7)	93%	96.9% (score ≥2.5)	92% (score 1)	78.0%
**Specificity**	75.3% (4 features of 7)	55%	76.6% (score ≥2.5)	46% (score 1)	93.4%
**Method applied for determination of MMR deficiency**	IHC (4 markers), *BRAF* mutation	MSI (10 markers) and IHC (4 markers); validation study: MSI (5 markers) and IHC (MLH1, MSH2), *BRAF* mutation	MSI (5 markers); validation cohort only 1 marker if age ≥75 years	MSI (4 markers)	MSI (11 markers)

*Abbreviations*: *HPF* high-power field, *IHC* immunohistochemistry, *LPF* low-power field, *MMR* mismatch repair, *MSI* microsatellite instability, *TIL* tumor-infiltrating lymphocytes.

## Methods

### Patients

All colon (n = 474) cancers from 462 patients (210 men and 252 women) who underwent surgery at the Helsingborg Hospital, Sweden between 2002 and 2006 were eligible for the study. None of the patients had a previous colorectal cancer diagnosis. In order to minimize the contribution from Lynch syndrome, patients diagnosed <50 years of age were excluded. Synchronous colon cancers were identified in 12 patients. The study was approved by the Lund University ethics committee.

### Histopathologic evaluation

All available hematoxylin and eosin (H&E) stained slides were morphologically evaluated according to a standardized protocol by two independent investigators (B.H. and P.J.) who were blinded to the immunohistochemistry results as well as to the results from the other reviewer. The evaluators considered invasive tumor components and did not take intramucosal/early invasive tumor components into account [8,26]. Tumor location was classified as proximal/distal in relation to the splenic flexure [2]. Tumor stage was determined according to the American Joint Cancer Committee/Union Internationale Contre le Cancer (AJCC/UICC) staging system and the grade according to the WHO system [27]. Mucinous/signet-ring cell cancers were considered poorly differentiated. Growth pattern was classified as expanding if a continuous, rounded infiltration margin was found and as infiltrating if invading foci were identified [10]. Dirty necrosis was defined as the presence of cell detritus and inflammatory cells within the glandular lumina and was scored as present or absent [9]. A tumor was classified as mucinous or signet-ring cell cancer if more than 50% of the tumor area showed such differentiation [27]. Tumors with mucinous/ signet-ring cell components that encompassed 10-50% of the area but did not fulfill the criteria for mucinous/signet-ring cell tumors were classified as having a mucinous/signet-ring cell component [25]. TIL were identified on H&E-stained slides and defined as intraepithelial lymphocytes between tumor cells; they were scored as present if there were ≥7 TIL per 10 high-power fields (40×, field diameter 0.53 mm) [8,26].

### Application of the MMR index

The MMR index includes the factors female sex, age ≥60 years, proximal tumor location, expanding growth pattern, lack of dirty necrosis, any mucinous/signet-ring cell differentiation (mucinous/signet-ring cell tumor or mucinous/signet-ring cell component) in ≥10% of the tumor area and presence of TIL. The index was applied to all tumors in the series. As previously reported [25], the presence of ≥4 factors was chosen as the cut-off limit based on optimal sensitivity and specificity. All slides were evaluated by B.H., In addition, 200 randomly selected tumors were independently evaluated by P.J. for the assessment of reproducibility. Complete data were obtained for 189 tumors, which were included in the final evaluation of inter-observer reproducibility.

### Immunohistochemical analysis

Fresh 4-μm sections were immunohistochemically stained using antibodies against MLH1 (clone G168-15, 1:50, BD PharMingen, San Diego, CA, USA or clone ES05, 1:100, Dako, Glostrup, Denmark), PMS2 (clone A16-4, 1:300, BD PharMingen), MSH2 (clone FE-11, 1:100, Calbiochem, La Jolla, CA, USA) and MSH6 (clone EPR3945, 1:100; Epitomics, Burlingame, CA, USA) using the EnVision™ (Dako, Glostrup, Denmark) detection kit [28]. MMR protein expression was evaluated without knowledge of the results of the morphologic review and was classified as retained (presence of nuclear staining) or lost (loss of nuclear staining with retained staining in stromal, inflammatory or non-neoplastic epithelial cells).

### Comparison with other predictive models

The MMR index results were compared with those from 5 other predictive models (Table 1), i.e. MsPath [19,23], PREDICT/simplified PREDICT [22], MSI probability score [21] and RERtest6 [20,24] in 200 randomly selected tumors, 20% (n = 40) of which were MMR deficient.

### Statistical analysis

For statistical calculations, the software package Stata 12.1 (StataCorp. 2012, College Station, TX, USA) was used. The histopathologic variables were dichotomized and assigned equal weights. The association between MMR status and the other histopathologic factors was analyzed by means of contingency tables and Fisher’s exact test. Patients with any missing value were excluded from the analysis (n = 24). A multiple logistic regression model that contained the 7 dichotomized clinicopathologic factors as covariates was fitted to determine the independent contribution of each factor at predicting MMR deficiency. These effects were summarized as odds ratios (OR) with 95% confidence intervals (CI). The sensitivity and specificity of the MMR index were calculated by means of a receiver operating characteristic (ROC) curve. Inter-observer variability was expressed using the chance-corrected measure of agreement kappa. The performance of the different models was evaluated by calculating the sensitivity, specificity, positive predictive value (PPV) and negative predictive value (NPV) and area under the ROC curve (AUC).

## Results

MMR deficiency, defined as immunohistochemical loss of at least one MMR protein, was identified in 108/474 (22.8%) tumors. No tumors showed weak or reduced MMR protein staining. The MMR deficient tumors predominantly developed in women (74.8%), in the proximal colon (91.7%) and were diagnosed at a mean age of 76 (range 50–100) years (Table 2). MMR defects involved MLH1/PMS2 in 93 tumors, PMS2 in 1, MSH2/MSH6 in 5, MSH6 in 4, and MLH1/PMS2 and MSH6 in 5. This means that defects highly suggestive of Lynch syndrome (mutations in *MSH2* and *MSH6*) were identified in 14/474 (3%) cases.

**Table 2:** Distribution of clinicopathologic factors in relation to MMR status (n = 474)

**Factor**		**Frequency (%)**			
		**MMR deficient**		**MMR proficient**	
**Patients (n = 462)**	Total number	103	(22.3)	359	(77.7)
**Sex (n = 462)**	Male	26	(25.2)	184	(51.3)
	Female	77	(74.8)	175	(48.7)
**Age (n = 462)**	Mean	76		74	
	Age ≥60	102	(99.0)	328	(91.4)
**Tumors (n = 474)**	Total number	108	(22.8)	366	(77.2)
**pT stage (n = 450)**	pT1	2	(1.9)	6	(1.7)
	pT2	10	(9.6)	37	(10.7)
	pT3	87	(83.7)	224	(64.8)
	pT4	5	(4.8)	79	(22.8)
**pN stage (n = 461)**	pN0	76	(73.1)	183	(51.2)
	pN1	23	(22.1)	107	(30.0)
	pN2	5	(4.8)	67	(18.8)
**pM stage (n = 474)**	pM1	1	(0.9)	10	(2.7)
**Differentiation (n = 474)**	Good/moderate	42	(38.9)	334	(91.3)
	Poor/no	66	(61.1)	32	(8.7)
**Location (n = 473)**	Proximal	99	(91.7)	173	(47.4)
	Distal	9	(8.3)	192	(52.6)
**Growth pattern (n = 459)**	Expanding	76	(73.8)	27	(7.6)
	Infiltrating	27	(26.2)	329	(92.4)
**Dirty necrosis (n = 467)**	Present	20	(19.4)	269	(73.9)
	Absent	83	(80.6)	95	(26.1)
**Mucin/signet-ring differentiation (n = 473)**	Present (>50%)	23	(21.3)	15	(4.1)
	Present (10-50%)	50	(46.3)	81	(22.2)
	Absent	35	(32.4)	269	(73.7)
**TIL (n = 470)**	Present	72	(66.7)	61	(16.9)
	Absent	36	(33.3)	301	(83.1)

*Abbreviations:MMR* mismatch repair, *TIL* tumor-infiltrating lymphocytes.

Several morphologic features were overrepresented in MMR deficient tumors in comparison with MMR proficient tumors (Table 2). This applied to expanding growth pattern (73.8% *versus* 7.6%), lack of dirty necrosis (80.6% *versus* 26.1%), mucinous/signet-ring cell differentiation (67.6% *versus* 26.3%) and presence of TIL (66.7% versus 16.9%). The strongest predictive indicators of MMR deficiency were expanding growth pattern (OR 11.6; 95% CI 5.5-24.5), presence of TIL (OR 5.6; 95% CI 2.6-12.1), mucinous/signet-ring cell differentiation (OR 3.0; 95% CI 1.3-6.0) and lack of dirty necrosis (OR 3.0; 95% CI 1.3-7.0). Inter-observer agreement was 90%, which corresponds to a kappa value of 0.88. The kappa values for the individual histopathologic markers were 0.78 for TIL, 0.94 for mucinous/signet-ring cell components, 0.96 for lack of dirty necrosis and 0.97 for expanding growth pattern.

**Figure 1:**
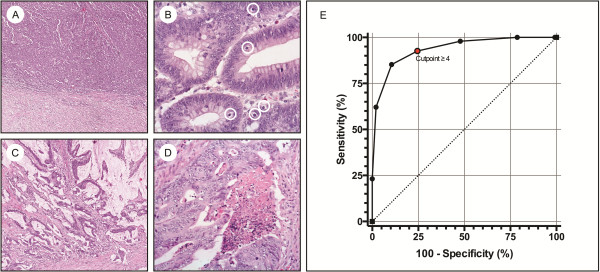
**Morphologic factors included in the MMR index. A)** Expanding growth pattern (x5), **B)** Tumor-infiltrating lymphocytes (x40), **C)** Mucinous differentiation (x10) and **D)** Dirty necrosis (x20). **E)** Receiver operating characteristic curve demonstrating sensitivity and specificity for an increasing number of factors in the index. Area under the curve 0.94. The suggested cut-off point (≥4 factors) is marked by a red dot.

The MMR index was applied in 438 patients from whom complete data were available. In these patients, the presence of ≥4 factors identified MMR deficient colon cancers with 92.6% sensitivity and 75.5% specificity, corresponding to an ROC curve with an AUC of 0.94 (95% CI, 0.91-0.96) (Figure 1). Comparison with other predictive models was performed in 200 randomly selected tumors, in which the MMR index – applied with a cut-off of ≥4 factors – resulted in an AUC of 0.83 (95% CI, 0.79-0.87) and identified MMR deficient tumors with 97.5% sensitivity and 69% specificity. The factors expanding growth pattern, TIL, mucinous/signet-ring cell differentiation and lack of dirty necrosis identified MMR deficient tumors with almost identical performance as identified in the original report of the MMR index [25]. The distribution of clinicopathologic features evaluated by the other models is supplementary table [see Additional file 1: Table S1]; the respective AUC values were 0.81 for PREDICT, 0.80 for RERtest6, 0.70 for MsPath and 0.77 for the MSI probability score. Sensitivities varied from 60% to 100% and specificities from 41% to 99% (Table 3). The performance of the MMR index was similar to that of the PREDICT/simplified PREDICT models (p = 0.38/p = 0.27) and the RERtest6 model (p = 0.42), but was significantly better than that of the MsPath model (p <0.0001) and the MSI probability score model (p <0.0001 for a cut-off >1 and p <0.01 for a cut-off >1.5).

**Table 3:** Performance of the different prediction models for MMR (n = 200)

**Model**	**Sensitivity (%)**	**Specificity (%)**	**PPV (%)**	**NPV (%)**	**AUC**
**MMR index**	97.5	68.8	44.3	99.1	0.83
**4 features present**					
**PREDICT**	95.0	66.3	41.3	98.2	0.81
**Score ≥2.5**					
**Simplified PREDICT**	95.0	65.0	40.4	98.1	0.80
**2 features present**					
**MsPath**	100.0	40.6	29.6	100.0	0.70
**Score ≥1**					
**MSI probability score**	100.0	45.6	31.5	100.0	0.73
**Score ≥1**	97.5	56.3	35.8	98.9	0.77
**Score ≥1.5**					
**RERtest6**	60.0	99.4	96.0	90.8	0.80
**Score <0.8**					

*Abbreviations:AUC* area under curve, *MMR* mismatch repair, *MSI* microsatellite instability, *NPV* negative predictive value, *PPV* positive predictive value.

## Discussion

The strongest predictive indicators of MMR deficiency were an expanding growth pattern (OR 11.6), presence of TIL (OR 5.6), mucinous/signet-ring cell differentiation (OR 3.0) and lack of dirty necrosis (OR 3.0) (Table 3). In comparison to the series in which the MMR index was established, the age groups studied differed somewhat, but the histopathologic characteristics were observed at similar frequencies, i.e. 59-89% in the former study and 67-81% in the present series [25]. The predictive values of the individual factors partly differed, but when combined into a MMR index, presence of ≥4 of the factors identified MMR defective tumors with similar sensitivity (93%) and specificity (75%) in the sample sets. Reproducibility was demonstrated through independent and blinded evaluation by two reviewers who identified MMR deficient tumors with a kappa value of 0.88. Hence, the model demonstrates stable performance, favorable prediction and is quick, cheap and easy to apply.

When compared with 5 other predictive models, the MMR index demonstrated better performance than the RERtest6 [20,24], MsPath [19,23] and the MSI probability score [21] models and comparable performance to the PREDICT/simplified PREDICT models [22] (Table 3). The MsPath, PREDICT and MSI probability scores all identified MMR deficient tumors with an equally favorable sensitivity, whereas the sensitivity of the RERtest6 model was clearly insufficient (60%) in our cohort. The MMR index identified 39/40 MMR deficient tumors and would have prompted the evaluation of MMR status in another 50 MMR proficient tumors. The number of MMR proficient tumors that would be included in testing with retained sensitivity was 54 for the PREDICT model, but it was considerably higher for the MSI probability score model (n = 70) and the MsPath (n = 95) model. With exception of the RERtest6 model, these algorithms have primarily been developed for the identification of Lynch syndrome tumors [21-23]. The MsPath, PREDICT and MSI probability score models therefore include age <50 years as one of the predictive variables, which means that comparison with the MMR index in our study was suboptimal given that the study only included cases diagnosed after age 50. The RERtest6 model was, however, developed without age restriction and has also been validated in independent tumor samples [20,24]. In our series, the RERtest6 model showed a high specificity but a low sensitivity; only 1 tumor that had been identified as MMR deficient was a false-positive, but 16/40 MMR deficient tumors escaped detection [see Additional file 1: Table S1].

MMR testing is commonly requested without prior selection in the 5% of colon cancers that develop before age 50 [29-31]. Our study thus focused on patients ≥50 years of age, 23% of whom had MMR deficient colon cancers with loss of MLH1/PMS2 in 86% of the cases. The consistent silencing of *MLH1* through promoter methylation has been suggested to lead to more pronounced histopathologic features than the variable MMR gene defects observed in Lynch syndrome tumors [6,15,32,33]. Despite our focus on a cohort over the age of 50, we identified immunohistochemical losses (of MSH2 and/or MSH6) suggestive of Lynch syndrome in 3% of the tumors. In particular, the contribution from MSH6 was substantial. *MSH6* mutations have been linked to an overall lower risk of colon cancer, less striking family history, higher age at onset and less pronounced tumor morphology, which implies that many cases may escape detection [34,35]. Indeed, the 4 cases with isolated loss of MSH6 and the 5 cases with combined loss of MLH1/PMS2 and MSH6 developed after age 70. Though these cases are not likely to influence the performance of the MMR index, the observation indicates that Lynch syndrome should be considered also among somewhat older patients. The MMR index is not intended for identification of Lynch syndrome cases and has not been validated in younger individuals. However, 11 of 14 MSH2/MSH6 deficient tumors in our series as well as all 7 MSH2/MSH6 deficient tumors reported by Halvarsson et al. [25] fulfilled the criteria and would have been detected by the MMR index.

## Conclusions

In summary, the MMR index provides a validated tool to identify the MMR deficient subset of colon cancers. Study limitations include assessment of MMR status solely based on immunohistochemical staining and inclusion of a small number of presumed Lynch syndrome tumors, which were not further genetically characterized. The impact from these shortcomings is judged to be minor and also mimics applicability in clinical routine. The MMR index does, in addition to factors evaluated as part of the routine diagnostic work-up (i.e. sex, age, tumor location and occurrence of mucinous differentiation) requires added evaluation only of growth pattern, dirty necrosis and TIL. The simple application and favorable reproducibility represent key advantages towards its clinical implementation for identification of individuals with a favorable prognosis who may be spared adjuvant chemotherapy.

## Competing interests

The authors declare that they have no competing interests.

## Authors’ contributions

PJ, POB, BH and MN participated in the design of the study. PJ and BH performed data acquisition and pathologic assessment. ER performed all immunohistochemical staining. POB and PJ performed the statistical analysis. PJ and MN drafted the manuscript. POB, BH and ER revised the manuscript. All authors read and approved the final manuscript.

## Pre-publication history

The pre-publication history for this paper can be accessed here:

http://www.biomedcentral.com/1472-6890/13/33/prepub

## Supplementary Material

Additional file 1: Table S1 Distribution of clinicopathologic features in the different prediction models for MMR (n = 200). Click here for file

## References

[B1] Ogino S Goel A Molecular classification and correlates in colorectal cancer J Mol Diagn 2008 13 1 13 27 10.2353/jmoldx.2008.070082 18165277 PMC2175539

[B2] Jass JR Do KA Simms LA Iino H Wynter C Pillay SP Searle J Radford-Smith G Young J Leggett B Morphology of sporadic colorectal cancer with DNA replication errors Gut 1998 13 5 673 679 10.1136/gut.42.5.673 9659163 PMC1727100

[B3] Kim H Jen J Vogelstein B Hamilton SR Clinical and pathological characteristics of sporadic colorectal carcinomas with DNA replication errors in microsatellite sequences Am J Pathol 1994 13 1 148 156 8030745 PMC1887287

[B4] Kakar S Burgart LJ Thibodeau SN Rabe KG Petersen GM Goldberg RM Lindor NM Frequency of loss of hMLH1 expression in colorectal carcinoma increases with advancing age Cancer 2003 13 6 1421 1427 10.1002/cncr.11206 12627505

[B5] Malkhosyan SR Yamamoto H Piao Z Perucho M Late onset and high incidence of colon cancer of the mutator phenotype with hypermethylated hMLH1 gene in women Gastroenterology 2000 13 2 598 10.1053/gast.2000.16154 10960275

[B6] Young J Simms LA Biden KG Wynter C Whitehall V Karamatic R George J Goldblatt J Walpole I Robin SA Features of colorectal cancers with high-level microsatellite instability occurring in familial and sporadic settings: parallel pathways of tumorigenesis Am J Pathol 2001 13 6 2107 2116 10.1016/S0002-9440(10)63062-3 11733361 PMC1850604

[B7] Lynch HT Smyrk TC Watson P Lanspa SJ Lynch JF Lynch PM Cavalieri RJ Boland CR Genetics, natural history, tumor spectrum, and pathology of hereditary nonpolyposis colorectal cancer: an updated review Gastroenterology 1993 13 5 1535 1549 8482467 10.1016/0016-5085(93)90368-m

[B8] Smyrk TC Watson P Kaul K Lynch HT Tumor-infiltrating lymphocytes are a marker for microsatellite instability in colorectal carcinoma Cancer 2001 13 12 2417 2422 10.1002/1097-0142(20010615)91:12<2417::AID-CNCR1276>3.0.CO;2-U 11413533

[B9] Greenson JK Bonner JD Ben-Yzhak O Cohen HI Miselevich I Resnick MB Trougouboff P Tomsho LD Kim E Low M Phenotype of microsatellite unstable colorectal carcinomas: well-differentiated and focally mucinous tumors and the absence of dirty necrosis correlate with microsatellite instability Am J Surg Pathol 2003 13 5 563 570 10.1097/00000478-200305000-00001 12717242

[B10] Jass JR Ajioka Y Allen JP Chan YF Cohen RJ Nixon JM Radojkovic M Restall AP Stables SR Zwi LJ Assessment of invasive growth pattern and lymphocytic infiltration in colorectal cancer Histopathology 1996 13 6 543 548 10.1046/j.1365-2559.1996.d01-467.x 8803598

[B11] Des Guetz G Schischmanoff O Nicolas P Perret GY Morere JF Uzzan B Does microsatellite instability predict the efficacy of adjuvant chemotherapy in colorectal cancer? a systematic review with meta-analysis Eur J Cancer 2009 13 10 1890 1896 10.1016/j.ejca.2009.04.018 19427194

[B12] Sargent DJ Marsoni S Monges G Thibodeau SN Labianca R Hamilton SR French AJ Kabat B Foster NR Torri V Defective mismatch repair as a predictive marker for lack of efficacy of fluorouracil-based adjuvant therapy in colon cancer J Clin Oncol 2010 13 20 3219 3226 10.1200/JCO.2009.27.1825 20498393 PMC2903323

[B13] Ribic CM Sargent DJ Moore MJ Thibodeau SN French AJ Goldberg RM Hamilton SR Laurent-Puig P Gryfe R Shepherd LE Tumor microsatellite-instability status as a predictor of benefit from fluorouracil-based adjuvant chemotherapy for colon cancer N Engl J Med 2003 13 3 247 257 10.1056/NEJMoa022289 12867608 PMC3584639

[B14] Jover R Zapater P Castells A Llor X Andreu M Cubiella J Balaguer F Sempere L Xicola RM Bujanda L The efficacy of adjuvant chemotherapy with 5-fluorouracil in colorectal cancer depends on the mismatch repair status Eur J Cancer 2009 13 3 365 373 10.1016/j.ejca.2008.07.016 18722765

[B15] Popat S Hubner R Houlston RS Systematic review of microsatellite instability and colorectal cancer prognosis J Clin Oncol 2005 13 3 609 618 15659508 10.1200/JCO.2005.01.086

[B16] Samowitz WS Curtin K Ma KN Schaffer D Coleman LW Leppert M Slattery ML Microsatellite instability in sporadic colon cancer is associated with an improved prognosis at the population level Cancer Epidemiol Biomarkers Prev 2001 13 9 917 923 11535541

[B17] Sinicrope FA Foster NR Thibodeau SN Marsoni S Monges G Labianca R Kim GP Yothers G Allegra C Moore MJ DNA mismatch repair status and colon cancer recurrence and survival in clinical trials of 5-fluorouracil-based adjuvant therapy J Natl Cancer Inst 2011 13 11 863 875 10.1093/jnci/djr153 21597022 PMC3110173

[B18] Parsons MT Buchanan DD Thompson B Young JP Spurdle AB Correlation of tumour BRAF mutations and MLH1 methylation with germline mismatch repair (MMR) gene mutation status: a literature review assessing utility of tumour features for MMR variant classification J Med Genet 2012 13 3 151 157 10.1136/jmedgenet-2011-100714 22368298

[B19] Bessa X Alenda C Paya A Alvarez C Iglesias M Seoane A Dedeu JM Abuli A Ilzarbe L Navarro G Validation microsatellite path score in a population-based cohort of patients with colorectal cancer J Clin Oncol 2011 13 25 3374 3380 10.1200/JCO.2010.34.3947 21788563

[B20] Colomer A Erill N Vidal A Calvo M Roman R Verdu M Cordon-Cardo C Puig X A novel logistic model based on clinicopathological features predicts microsatellite instability in colorectal carcinomas Diagn Mol Pathol 2005 13 4 213 223 10.1097/01.pas.0000177800.65959.48 16319691

[B21] Greenson JK Huang SC Herron C Moreno V Bonner JD Tomsho LP Ben-Izhak O Cohen HI Trougouboff P Bejhar J Pathologic predictors of microsatellite instability in colorectal cancer Am J Surg Pathol 2009 13 1 126 133 10.1097/PAS.0b013e31817ec2b1 18830122 PMC3500028

[B22] Hyde A Fontaine D Stuckless S Green R Pollett A Simms M Sipahimalani P Parfrey P Younghusband B A histology-based model for predicting microsatellite instability in colorectal cancers Am J Surg Pathol 2010 13 12 1820 1829 10.1097/PAS.0b013e3181f6a912 21107088

[B23] Jenkins MA Hayashi S O’Shea AM Burgart LJ Smyrk TC Shimizu D Waring PM Ruszkiewicz AR Pollett AF Redston M Pathology features in Bethesda guidelines predict colorectal cancer microsatellite instability: a population-based study Gastroenterology 2007 13 1 48 56 10.1053/j.gastro.2007.04.044 17631130 PMC2933045

[B24] Roman R Verdu M Calvo M Vidal A Sanjuan X Jimeno M Salas A Autonell J Trias I Gonzalez M Microsatellite instability of the colorectal carcinoma can be predicted in the conventional pathologic examination: a prospective multicentric study and the statistical analysis of 615 cases consolidate our previously proposed logistic regression model Virchows Arch 2010 13 5 533 541 10.1007/s00428-010-0896-6 20393748

[B25] Halvarsson B Anderson H Domanska K Lindmark G Nilbert M Clinicopathologic factors identify sporadic mismatch repair-defective colon cancers Am J Clin Pathol 2008 13 2 238 244 10.1309/0PP5GDRTXUDVKAWJ 18208804

[B26] Jass JR Role of the pathologist in the diagnosis of hereditary non-polyposis colorectal cancer Dis Markers 2004 13 4–5 215 224 15528787 10.1155/2004/197484 PMC3839333

[B27] Hamilton SR Bosman FT Boffetta P Ilyas M Morreau H Nakamura SI Quirke P Riboli E Sobin LH Bosman FT, Carneiro F, Hruban RH, Theise ND Carcinoma of the colon and rectum WHO classification of tumours of the digestive system: 3rd volume 2010 4 Lyon: IARC Press 134 146

[B28] Halvarsson B Lindblom A Rambech E Lagerstedt K Nilbert M Microsatellite instability analysis and/or immunostaining for the diagnosis of hereditary nonpolyposis colorectal cancer? Virchows Arch 2004 13 2 135 141 10.1007/s00428-003-0922-z 14652751

[B29] Manders P Spruijt L Kets CM Willems HW Bodmer D Hebeda KM Nagtegaal ID van Krieken JH Ligtenberg MJ Hoogerbrugge N Young age and a positive family history of colorectal cancer are complementary selection criteria for the identification of Lynch syndrome Eur J Cancer 2011 13 9 1407 1413 10.1016/j.ejca.2010.12.024 21273057

[B30] Steinhagen E Shia J Markowitz AJ Stadler ZK Salo-Mullen EE Zheng J Lee-Kong SA Nash GM Offit K Guillem JG Systematic immunohistochemistry screening for Lynch syndrome in early age-of-onset colorectal cancer patients undergoing surgical resection J Am Coll Surg 2012 13 1 61 67 10.1016/j.jamcollsurg.2011.10.004 22192923

[B31] Schofield L Grieu F Goldblatt J Amanuel B Iacopetta B A state-wide population-based program for detection of lynch syndrome based upon immunohistochemical and molecular testing of colorectal tumours Fam Cancer 2012 13 1 1 6 10.1007/s10689-011-9494-2 22120844

[B32] Jass JR Walsh MD Barker M Simms LA Young J Leggett BA Distinction between familial and sporadic forms of colorectal cancer showing DNA microsatellite instability Eur J Cancer 2002 13 7 858 866 10.1016/S0959-8049(02)00041-2 11978509

[B33] Gryfe R Kim H Hsieh ET Aronson MD Holowaty EJ Bull SB Redston M Gallinger S Tumor microsatellite instability and clinical outcome in young patients with colorectal cancer N Engl J Med 2000 13 2 69 77 10.1056/NEJM200001133420201 10631274

[B34] Klarskov L Holck S Bernstein I Okkels H Rambech E Baldetorp B Nilbert M Challenges in the identification of MSH6-associated colorectal cancer: rectal location, less typical histology, and a subset with retained mismatch repair function Am J Surg Pathol 2011 13 9 1391 1399 10.1097/PAS.0b013e318225c3f0 21836479

[B35] Wagner A Hendriks Y Meijers-Heijboer EJ de Leeuw WJ Morreau H Hofstra R Tops C Bik E Brocker-Vriends AH van Der Meer C Atypical HNPCC owing to MSH6 germline mutations: analysis of a large Dutch pedigree J Med Genet 2001 13 5 318 322 10.1136/jmg.38.5.318 11333868 PMC1734864

