# Supplementary material for: Efficient and reproducible identification of mismatch repair deficient colon cancer: validation of the MMR index and comparison with other predictive models
Source: BMC Clin Pathol. 2013 Dec 17;13:33. doi: 10.1186/1472-6890-13-33 (PMC3878549; doi:10.1186/1472-6890-13-33)
Supplement: Additional file 1: Table S1 — Distribution of clinicopathologic features in the different prediction models for MMR (n = 200). [file 1472-6890-13-33-S1.pdf]

Supplementary table:
Distribution of clinicopathologic features in the different prediction models for MMR (n = 200).

Prediction model	Factor	Frequency (%)	
		MMR deficient	MMR proficient
	Total number of patients/tumors	40 (20.0)	160 (80.0)
	Mean age	77	76
**MMR index**	Female sex	28 (70.0)	83 (51.9)
	Age ≥60	40 (100.0)	160 (100.0)
	Proximal location	37 (92.5)	78 (48.8)
	Expanding growth pattern	28 (70.0)	17 (10.6)
	Lack of dirty necrosis	35 (87.5)	49 (30.6)
	≥10% mucinous/signet-ring differentiation	24 (60.0)	44 (27.5)
	TIL (≥7/10 HPF)	34 (85.0)	33 (20.6)
	≥4 factors	39 (97.5)	50 (31.3)
**MsPath**	Age <50	0 (0.0)	0 (0.0)
	Proximal location	37 (92.5)	78 (48.8)
	Mucinous, signet ring or undifferentiated carcinoma	11 (27.5)	7 (4.4)
	TIL (≥5/HPF, 10 searched)	31 (77.5)	26 (16.3)
	Poor differentiation	32 (80.0)	51 (31.9)
	Crohn-like reaction (≥4 nodules/LPF)	19 (47.5)	35 (21.9)
	MsPath score ≥1	40 (100.0)	95 (59.4)
**PREDICT**	Age ≤50	0 (0.0)	0 (0.0)
	Proximal location	37 (92.5)	78 (48.8)
	Any mucinous differentiation	26 (65.0)	54 (33.8)
	TIL (≥5/HPF, 10 searched)	31 (77.5)	26 (16.3)
	Peritumoral reaction	11 (27.5)	23 (14.4)
	Increased stromal plasma cells	3 (7.5)	7 (4.4)
	PREDICT score ≥2.5	38 (95.0)	54 (33.8)
**Simplified PREDICT**	≥2 features present	38 (95.0)	56 (35.0)
**MSI probability score**	Age ≤50	0 (0.0)	0 (0.0)
	Proximal location	37 (92.5)	78 (48.8)
	Lack of dirty necrosis	35 (87.5)	49 (30.6)
	Any mucinous differentiation	26 (65.0)	54 (33.8)
	TIL (≥2/HPF, mean of 5 searched)	34 (85.0)	34 (21.3)
	Well and poorly differentiated	32 (80.0)	52 (32.5)
	(≥3 nodules/section)	24 (60.0)	74 (46.3)
	Score ≥1	40 (100.0)	87 (54.4)
	Score ≥1.5	39 (97.5)	70 (45.8)
**RERtest6**	Proximal location	37 (92.5)	78 (48.8)
	Expanding growth pattern	28 (70.0)	17 (10.6)
	Mucinous pattern in % (mean)	24.1	8.2
	Solid pattern in % (mean)	19.4	1.8
	TIL (≥4/HPF)	34 (85.0)	29 (18.1)
	(≥3 nodules/LPF)	22 (55.0)	42 (26.3)
	RERtest6 result <0.8	24 (60.0)	1 (0.6)

Abbreviations: HPF, high-power field; LPF, low-power field; MMR, mismatch repair; TIL, tumor-infiltrating lymphocytes.
